# Effectiveness of routine provision of feedback from patient‐reported outcome measurements for cancer care improvement: a systematic review and meta-analysis

**DOI:** 10.1186/s41687-023-00578-8

**Published:** 2023-06-05

**Authors:** Sheng-Chieh Lu, I. Porter, J. M. Valderas, C. J. Harrison, Chris Sidey-Gibbons

**Affiliations:** 1 Department of Symptom Research, The University of Texas MD Anderson Cancer Center, 6565 MD Anderson Blvd., Houston, TX 77030 USA; 2 College of Medicine and Health, University of Exeter, Exeter, UK; 3 Department of Family Medicine, National University Health System, Singapore, Singapore; 4 Centre for Health Services Research, National University Health System, Singapore, Singapore; 5 Division of Family Medicine, Yong Loo Lin School of Medicine, National University of Singapore, Singapore, Singapore; 6 Nuffield Department of Orthopedics, Rheumatology and Musculoskeletal Sciences, University of Oxford, Oxford, UK

**Keywords:** Patient-reported outcome measure, Patient-centered care, Cancer care, Systematic review and meta-analysis

## Abstract

**Background:**

Research shows that feeding back patient-reported outcome information to clinicians and/or patients could be associated with improved care processes and patient outcomes. Quantitative syntheses of intervention effects on oncology patient outcomes are lacking.

**Objective:**

To determine the effects of patient-reported outcome measure (PROM) feedback intervention on oncology patient outcomes.

**Data sources:**

We identified relevant studies from 116 references included in our previous Cochrane review assessing the intervention for the general population. In May 2022, we conducted a systematic search in five bibliography databases using predefined keywords for additional studies published after the Cochrane review.

**Study selection:**

We included randomized controlled trials evaluating the effects of PROM feedback intervention on processes and outcomes of care for oncology patients.

**Data extraction and synthesis:**

We used the meta-analytic approach to synthesize across studies measuring the same outcomes. We estimated pooled effects of the intervention on outcomes using Cohen’s d for continuous data and risk ratio (RR) with a 95% confidence interval for dichotomous data. We used a descriptive approach to summarize studies which reported insufficient data for a meta-analysis.

**Main outcome(s) and measures(s):**

Health-related quality of life (HRQL), symptoms, patient-healthcare provider communication, number of visits and hospitalizations, number of adverse events, and overall survival.

**Results:**

We included 29 studies involving 7071 cancer participants. A small number of studies was available for each metanalysis (median = 3 studies, ranging from 2 to 9 studies) due to heterogeneity in the evaluation of the trials. We found that the intervention improved HRQL (Cohen’s d = 0.23, 95% CI 0.11–0.34), mental functioning (Cohen’s d = 0.14, 95% CI 0.02–0.26), patient-healthcare provider communication (Cohen’s d = 0.41, 95% CI 0.20–0.62), and 1-year overall survival (OR = 0.64, 95% CI 0.48–0.86). The risk of bias across studies was considerable in the domains of allocation concealment, blinding, and intervention contamination.

**Conclusions and relevance:**

Although we found evidence to support the intervention for highly relevant outcomes, our conclusions are tempered by the high risk of bias relating mainly to intervention design. PROM feedback for oncology patients may improve processes and outcomes for cancer patients but more high-quality evidence is required.

**Supplementary Information:**

The online version contains supplementary material available at 10.1186/s41687-023-00578-8.

## Introduction

Patient-reported outcomes can be broadly defined as any reports directly from patients about any aspect of their health or wellbeing without interpretation by others, including healthcare providers [1]. Patient-reported outcome measures (PROMs) are standardized and validated tools to collect a variety of outcomes, including health-related quality of life (HRQL), symptom severity, and treatment satisfaction [2–6]. PROMs have been used as tools to assess outcomes in clinical trials for many years [7, 8]. Alongside their use in research studies, there is growing enthusiasm to use PROMs in clinical practice to identify and quantify unmet needs and monitor outcomes [2, 4, 9, 10].

Cancer patients often experience various treatment-related symptoms [11]. Suboptimal management of these symptoms contributes to higher healthcare use and poorer outcomes including reductions in patient functioning, quality of life, and survival [3, 4, 12]. Use of PROMs enables early identification of symptoms and may facilitate timely provision of interventions to improve symptom management [13]. As professional groups and policy initiatives keep promoting the utilization of the PROM feedback intervention in oncology practices, several PROM feedback interventions have been developed and shown to be effective in improving the process and outcomes of oncology care [4, 7, 14, 15].

Synthesized evidence suggested that feeding back PRO information to clinicians and/or patients is associated with improved symptom identification, patient satisfaction, and patient-healthcare provider communication for cancer patients and care [3, 4, 12, 16]. However, the effectiveness of the intervention on the improvements in several outcomes, including HRQL and survival, is not clear. Previous systematic reviews consistently indicate that the quality concerns surrounding PROM feedback intervention trials may obfuscate true effects [4, 16]. There is also a lack of meta-analyses to quantitatively evaluate the impacts of the intervention on oncology care and outcomes [16].

The objective of this study was to quantitatively synthesize current evidence relating to the effects of the PROM feedback intervention on processes and outcomes for oncology care. Specifically, we examined patient outcomes, including HRQL, functioning, a variety of common symptoms for cancer patients, overall survival (OS), and treatment-related adverse events (AEs). We also examined the impact of PROM feedback on communication between patients and healthcare providers and use of services (visits and unplanned hospitalizations).

## Methods

This work follows a recently-published Cochrane review assessing the effects of PROM feedback interventions on processes of care and patient-reported outcomes. Detailed methods have been described elsewhere [2]. In summary, we followed the Cochrane guideline for systematic review of interventions [17] to conduct literature search, data extraction, and evidence evaluation and synthesis as described in the sequential sections. In the current study, we opted to include one additional outcome of OS to reflect the changing remit of PROM feedback interventions in oncology.

### Search strategy and study selection

In this study, the 116 references included in the Cochrane review was the major source we used to identify relevant studies. Detailed strategies for identification and inclusion of the 116 references have been documented in the publication [2]. To focus on oncology patients, we applied two criteria to select studies from the 116 studies: (1) recruited oncology participants in primary or secondary/tertiary care settings, and (2) was a full paper published in a peer-reviewed journal. Two researchers independently assess the title and abstracts of all 116 studies using the criteria. Studies rated as relevant by at least one reviewer were further independently reviewed in full-text by two researchers. We included studies rated as relevant during the full-text screen by all reviewers. We resolved discrepancies among reviewers through consensus.

To obtain studies published after October 2020, we conducted additional search in MEDLINE, EMBASE, CINAHL, PsycINFO, and Cochrane database using the same search strategy documented in the Cochran review with extra keywords, including cancer, oncology, tumor, and neoplasm, on May 2, 2022. We used the same eligibility and study selection strategy described previously to select relevant studies identified from the additional search. We provided the search strategy for each database in Additional file 1: eMethods.

### Data extraction

We collated data and assessed outcomes including health-related quality of life (HRQL), functioning (physical, mental, and social), symptoms (anorexia, anxiety, constipation, cough, depression, diarrhea, dyspnea, fatigue, insomnia, nausea, and pain), OS, patient-healthcare provider communication, use of services (numbers of visits and unplanned hospitalizations), and number of adverse events (AEs). We selected these outcomes because they are important outcomes indicating the quality of oncology care and widely used indicators for the effectiveness of PROM feedback interventions.

### Risk of bias assessment

We assessed the risk of bias (ROB) of the included studies using the Risk of Bias (RoB 1) tool with additional items suggested by the Cochrane Effective Practice and Organization of Care group [2, 18]. The tool covers nine domains: random sequence generation; allocation concealment; blinding of participants; blinding of outcome assessment; similarity of baseline measurement; incomplete outcome data; protection against contamination; and selective reporting; and other sources of bias [19, 20].

### Data synthesis and analysis

Due to the variety of outcome measures reported, we used either a quantitative meta-analytic approach to synthesize results across studies measuring same outcomes or a descriptive approach to summarize the size and direction of intervention effect for each study which reported insufficient data for inclusion in a meta-analysis. In quantitative analysis, we calculated Cohen’s d for continuous data and risk ratio (RR) with 95% confidence interval for dichotomous data. We then estimated pooled effects of the intervention on outcomes using random-effect models and evaluated heterogeneity among studies using the I^2^ statistic (I^2^ > 50% representing a substantial heterogeneity problem) [21]. We conducted data synthesis using RevMan 5 [18] and STATA v13 [22].

## Results

### Study selection and characteristics

**Fig. 1:**
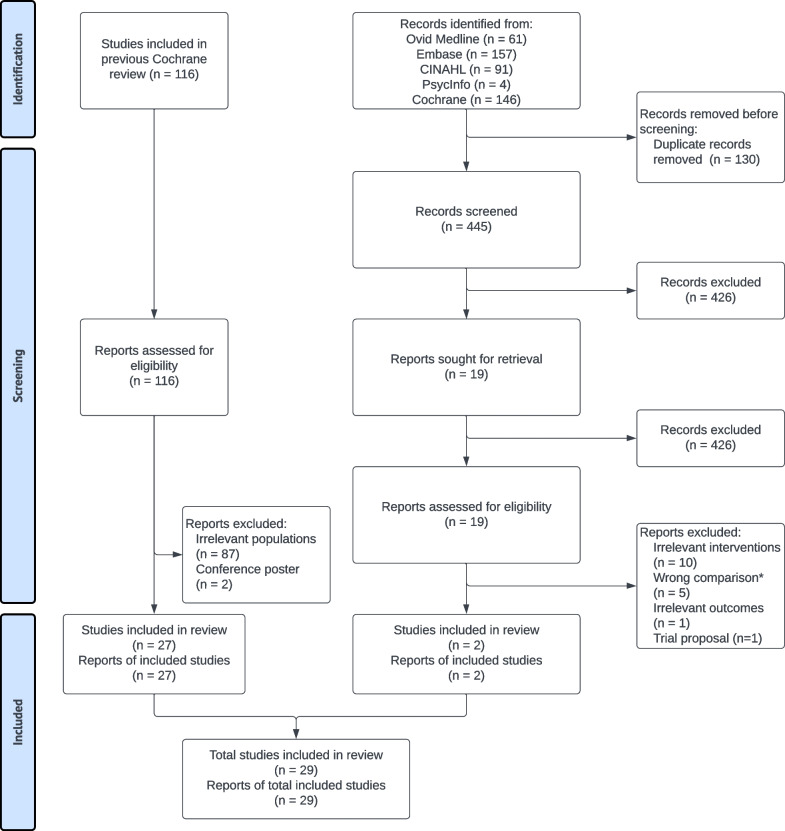
PRISMA flowchart for study selection. *Note*: *Both groups accessed PROM interventions in the studies

We included 27 oncology studies identified in the previous Cochrane review and additional two from the updating search (Fig. 1). We provide the characteristics of all 29 studies in Table 1 [15, 23–53]. The majority of the studies were conducted in high-income countries, including the United States (n = 14) [24–27, 30, 33, 38–41, 44, 46, 47, 52], the Netherlands (n = 4) [29, 32, 45, 48], United Kingdom (n = 3) [15, 23, 37], Australia (n = 3) [31, 35, 36], China (n = 1) [50], Canada (n = 1) [34], France (n = 1) [28], Denmark (n = 1) [43], and Switzerland (n = 1) [42]. All included studies were written in English.

**Table 1:** Characteristics of the included studies

Study	Population	Design	Participants			Outcome measures
			N	Age (mean & SD)	Sex (female %)	
Absolom [23] UK	Adult patients initiating chemotherapy for colorectal, breast, or gynecological cancers	Two-arm parallel RCT	Txt: 256 Ctrl: 252	Txt: 55.9 (12.2) Ctrl: 56.0 (11.3)	Txt: 80.1% Ctrl: 79.8%	FACT-PWB, Hospital services and cost-effectiveness using EHR data, Self-Efficacy Scale for managing chronic disease questionnaire, EQ-5D-5, EQ-5D-VAS, QLU-C10D, FACT-G, EORTC QLQ-C30
Anderson [24] USA	Low-income African American and Latina breast cancer patients	RCT	Txt: 31 Ctrl: 29	Txt: 49.6 (9.9) Ctrl: 50.5 (11.0)	Txt: 100% Ctrl: 100%	MDASI, BQ-II, Pain management index
Basch [25] USA	Adult patients initiating chemotherapy	RCT	Txt: 441 Ctrl: 325	All: median: 61 (26 – 91)	All: 58%	EuroQol EQ-5D Index, 1-year OS, number of ED visits
Bryant [26] USA	Oncology adult patients scheduled inpatient care following bone marrow transplant	RCT	Txt: 38 Ctrl: 38	Txt: 51.3 (13.6) Ctrl: 51.1 (13.7)	Txt: 78.9% Ctrl: 68.4%	PRO-CTCAE survey, HCT-CI
Cleeland [27] USA	Adult patients receiving thoracotomy for lung cancer or lung metastasis	RCT	Txt: 50 Ctrl: 50	Txt: 59.2 (13.6) Ctrl: 60.9 (11.8)	Txt: 44.7% Ctrl: 48.8%	MDASI, satisfaction with postoperative symptom control
Denis [28] France	Adult patients with advanced lung cancer	Multi-center RCT	Txt: 66 Ctrl: 67	Median (range) Txt: 65 (36 – 87) Ctrl: 64 (43 – 88)	Txt: 31.7% Ctrl: 34.4%	OS, PFS, FACT-L, number of unscheduled visits
Detmar [29] The Netherlands	Adult patients receiving palliative chemotherapy	Cluster RCT	Txt: 114 Ctrl: 200	Txt: 58 (NR) Txt: 55 (NR)	Txt: 73% Ctrl: 81%	COOP, WONCA, 5-item Patient Satisfaction Questionnaire, SF-36
Fann [30] USA	Adult patients initiating cancer therapy	RCT	Txt: 289 Ctrl: 292	Median (range) Txt: 56 (33–86) Ctrl: 59 (19–88)	Txt: 50% Ctrl: 46%	PHQ-9, QLQ-C30
Girgis [31] Australia	Adult patients with nonlocalized breast or colorectal cancer within 6 months of diagnosis	RCT	Txt 1: 119 Txt 2: 120 Ctrl: 117	Txt 1: 58.3 Txt 2: 57.8 Ctrl: 57.4	Txt 1: 72.3% Txt 2: 72.5% Ctrl: 71.8	HADS, EORCT, 34-item Supportive Needs Survey – Short Form, 10 items from the Needs Assessment for Advanced Cancer Patient Questionnaire, One question for perceived improvement in patient-physician communication
Hoekstra [32] The Netherlands	Adult patients with cancer in the palliative phase	Cluster RCT	Txt: 69 Ctrl: 77	Txt: 64.1 (NR) Ctrl: 64.6 (NR)	Txt: 53.6% Ctrl: 58.4%	Symptom Monitor (assessing 10 symptoms) self-report instrument
Kornblith [33] USA	Older adult patients with advanced breast, prostate, and colorectal cancers	RCT	Txt: 69 Ctrl: 62	Txt: 73 (5.7) Ctrl: 74 (6.8)	Txt: 48% Ctrl: 47%	EORTC QLQ-30, GDS short form, HADS, MOS Social Support Survey, the Older American Resources and Services Questionnaire Physical Health subscale
Kuo [34] Canada	Adult patients with incurable NSCLC	RCT	Txt: 33 Ctrl: 51	Median (range) Txt: 63 (43 – 80) Ctrl: 67 (39 – 80)	Txt: 43% Ctrl: 45%	Palliative referral rate, number of first-line chemotherapy cycles administered, referral to and use of other supportive interventions, changes in HRQL
Lugtenberg [48] The Netherlands	Adult patients with early-stage breast cancer (stage I-III) receiving chemotherapy	RCT	Txt: 60 Ctrl: 53	Txt: 51 (10.9) Ctrl: 52.1 (9.6)	Txt: 100% Ctrl: 100%	EORTC-QLQ C30, BIPQ, PEPPI, MCQ-C, NCCD DT, HADS
McLachlan [35] Australia	Adult oncology patients from ambulatory clinics	RCT	Txt: 296 Ctrl: 154	Median (range) 61 (18–92)	49%	Changes in CNQ, EORTC QLQ-C30, and BDI-SF
Moore [36] Australia	Adult patients with a new diagnosis of multiple myeloma	Parallel RCT	32	Median (range) Txt: 66 (59 – 76) Ctrl: 69 (62 – 71)	NR	Myeloma Patient Outcome Scale
Nimako [37] UK	Adult patients receiving cancer treatment	RCT	Txt: 45 Ctrl 1: 47 Ctrl 2: 46	Median (range) Txt: 66 (32—80) Ctrl 1: 66 (19—83) Ctrl 2: 64 (35 – 85)	Txt: 44% Ctrl 1: 45% Ctrl 2: 46%	EORTC QLQ-C30, EORTC QLQ-LC13, the number of QoL issues identified, the number of management actions, the number of contacts outside of clinics
Nipp [38] USA	Adult patients with a diagnosis of advanced cancer receiving inpatient oncology services	RCT	Txt: 75 Ctrl: 75	Txt: 60.4 (14.6) Ctrl: 64.9 (12.4)	Txt: 40.0% Ctrl: 41.3%	ESAS, PHQ-4, hospital length of stay, unplanned readmission within 30 and 90 days of hospital discharge
Nipp [52] USA	Adult patients with a diagnosis of advanced cancer	RCT	Txt: 160 Ctrl: 161	Txt: 64.5 (12.4) Ctrl: 62.7 (13.1)	Txt: 43.8% Ctrl: 44.1%	ESAS, PHQ-4, hospital length of stay, unplanned readmission within 30 and 90 days of hospital discharge
Rosenbloom [39] USA	Adult patients with metastatic breast, lung, or colorectal cancers	Cluster RCT	Txt 1: 73 Txt 2: 69 Ctrl: 71	Txt 1: 60.2 (11.0) Txt 2: 57.3 (11.8) Ctrl: 60.6 (9.3)	Txt 1: 30% Txt 2: 33% Ctrl: 36%	FLIC, Brief POMS-17, PSQ-III, author-developed clinical treatment change assessment tool
Ruland (2003) Norway	Adult oncology patients receiving treatment in outpatient clinics	Cluster RCT	Txt: 27 Ctrl: 25	56.3 (11.3)	59%	CHOICE, time requirement to complete the assessment, Ease of Use scale, 12-item Patient Satisfaction with Decision Making questionnaire
Ruland [40] USA	Adult patients initiating treatment for leukemia or lymphoma	RCT	Txt: 75 Ctrl: 70	Txt: 50 (15) Ctrl: 49 (15)	Txt: 40% Ctrl: 36%	Changes in symptom distress and changes in patients’ needs using an author-developed assessment tool
Strasser [42] Switzerland	Adult patients initiating outpatient chemotherapy with palliative intentions for incurable, symptomatic solid tumors	Cluster RCT	Txt: 119 Ctrl: 145	Median (range) Txt: 65 (40—84) Ctrl: 67 (35—84)	Txt: 51% Ctrl: 47%	EORTC-QLQ-C30, ESAS, patient-physician communication using a validated scale, KPS
Tolstrup [43] Denmark	Adult patients initiating immune checkpoint inhibitor treatments for unresectable stage III or IV disease	RCT	Txt: 73 Ctrl: 73	Median (range) Txt: 66 (34 – 87) Ctrl: 66 (32 – 83)	Txt: 52% Ctrl: 41%	CTCAE for changes in adverse event frequency and severity, number of extra outpatient visits
Trowbridge [44] USA	Adult cancer patients with oncologic pain	RCT	Txt: 260 Ctrl: 250	Median (range) Txt: 65.6 (18 – 92) Ctrl: 65.8 (21 – 91)	Txt: 57% Ctrl: 46%	Pain management index
Velikova [15] UK	Adult cancer patients	RCT	Txt: 144 Ctrl 1: 70 Ctrl 2: 72	Txt: 55.1 (13.0) Ctrl 1: 54.8 (12.5) Ctrl 2: 54.7 (11.7)	Txt: 75% Ctrl 1: 70% Ctrl 2: 73%	FACT-G
van der Hout [45] The Netherlands	Adult patients with a diagnosis of lymphoma, and head and neck, colorectal, and breast cancers	RCT	Txt: 320 Ctrl: 305	Median (range) Txt: 65 (56 – 71) Ctrl: 65 (57 – 71)	Txt: 49% Ctrl: 52%	Patient activation measure, EORTC QLQ-C30, supportive care needs, general self-efficacy scale, Pearlin and Schooler mastery scale, perceived efficacy patient-physician interactions scale
Wheelock [46] USA	Adult patients with TNM stage I to III breast cancer	RCT	Txt: 59 Ctrl: 41	Txt: 54.8 (8.7) Ctrl: 53.3 (10.8)	Txt: 100% Ctrl: 100%	Time in days between symptom reporting and remote valuation of symptoms, number of breast cancer-related visits, medical appointments, lab and image studies
Wolfe [47] USA	Pediatric cancer patients	Parallel RCT	Txt: 51 Ctrl: 53	Txt: 68% > = 8 years old Ctrl 69% > = 8 years old	Txt: 51% Ctrl: 47%	PQ-MSAS, PedsQL4.0, Sickness scores
Zhang [51] China	Adult patients receiving cancer immunotherapy	RCT	Txt: 141 Ctrl: 137	Txt: 57.6 (12.6) Ctrl: 60.1 (12.7)	Txt: 24.8% Ctrl: 27.0%	Rate of occurrence of grade 3 or 4 irAEs, ED visits, rate of treatment discontinuation and death owing to irAEs, QLQ-C30

*BDI – SF*, Beck depression inventory – short form; *BIPQ*, the brief illness perception questionnaire; *BPI*, brief pain Inventory; *BQ-II*, the barriers questionnaire II; *CHOICE*, creating better health outcomes by improving communication about patients’ experiences; *CNB*, the care notebook; *CNQ*, care needs questionnaire – short form; *COOP*, Dartmouth primary care cooperative information functional health assessment; *Ctrl*, control group; *ED*, emergency department; *EHR*, electronic health record; *eLCSS-QL*, the electronic lung cancer symptom scale; *EORTC QLQ-C30*, the European Organization for Research and Treatment of Cancer quality of life questionnaire C30; *EORTC QLQ-LC13*, the European Organization for Research and Treatment of Cancer quality of life questionnaire – lung cancer 13; *EQ-5D*-*5*, five level version of EuroQol five-dimensional; *EQ-5D-VAS*, EuroQol five-dimensional using visual analogue scale; *ESAS*, Edmonton symptom assessment system; *ESRA-C*, electronic self-report assessment for cancer; *FACT-G*, the functional assessment of cancer therapy – general; *FACT-L*, the functional assessment of cancer therapy – lung; *FACT-PWB*, the functional assessment of cancer therapy – physical and well-being; *FLIC*, functional living index – cancer; *HADS*, hospital anxiety and depression scale; *HCT-CI*, hematopoietic cell transplantation-comorbidity index; *HRQL*, health-related quality of life; *irAE*, immune-related adverse events; *IVR*, interactive voice response; *KPS*, the Karnofsky performance scale index; *MCQ-C*, medical care questionnaire – communication; *MDASI*, MD Anderson Symptom Inventory; *MOS*, medical outcomes study; *NCCN DT*, national comprehensive cancer network distress thermometer; *NR*, not reported; *NSCLC*, non-small cell lung cancer; *OS*, overall survival; *PC*, personal computer; *PEDsQL4.0*, the pediatric quality of life inventory 4.0 generic core scales; *PEPPI*, perceived efficacy in patient – physician interactions; *PFS*, progression-free survival; *PHQ*, patient health questionnaire; *POMS-17*, profile of mood states – 17; *PQ-MSAS*, the PediQUEST memorial symptom assessment scale; *PRO-CTCAE*, patient-reported outcome version of the common terminology criteria for adverse events; *PSQ-III*, patient satisfaction questionnaire – III; *QLU-C10D*, the EORTC quality of life utility measure-Core 10 dimensions; *RCT*, randomized controlled trial; *SF-36*, 36-item short-form health survey; *Txt*, treatment group; *WONCA*, World Organization Project of National Colleges and Academics

### Risk of bias assessment

**Fig. 2:**
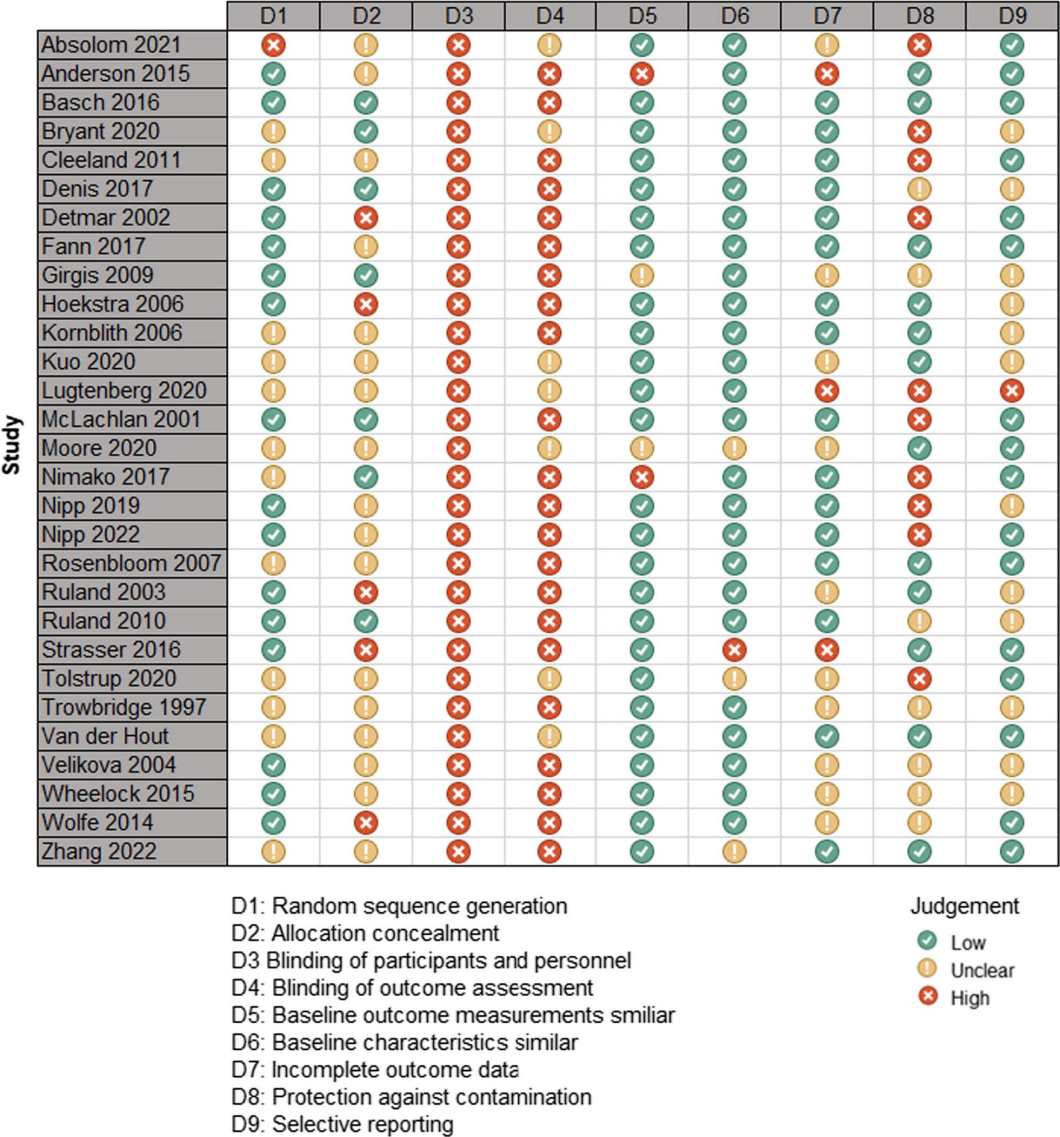
Summary of risk of bias assessment results

We summarized the results of our ROB assessment in Fig. 2. Overall, risk of bias across studies was considerable. We rated random sequence generation as high ROB for one study [23] and as unclear ROB for 12 studies [26, 27, 33, 34, 36, 37, 39, 43–45, 48, 50]. We found inappropriate allocation concealment in five studies [29, 32, 41, 42, 47] and missing allocation disclosure in 17 studies [15, 23, 24, 27, 30, 33, 34, 36, 38, 39, 43–46, 48, 50, 52]. It was not feasible for all studies to blind their participants and personnel due to the nature of the interventions, and thus we rated all studies as high ROB for this criterion. Similarly, we assessed blinding of outcome assessment as high ROB for 22 studies [15, 24, 25, 27–33, 35, 37–42, 44, 46, 47, 50, 52]. We did not have enough information for seven studies [23, 26, 34, 36, 43, 45, 48] and rated those studies as unclear ROB of detection bias. We assessed between-group differences in baseline characteristics as high ROB for three studies [24, 37, 42] and as unclear ROB for four studies [31, 36, 43, 50]. We found three studies [24, 42, 48] suffered from attrition bias due to the use of inappropriate strategies for addressing missing data. We assessed attrition bias as unclear ROB for 10 studies [15, 23, 31, 34, 36, 41, 43, 44, 46, 47]. For risk of intervention contamination, high ROB was evident in 10 studies [23, 26, 27, 29, 35, 37, 38, 43, 48, 52], and we assessed seven studies [15, 28, 31, 40, 44, 46, 47] to have an unclear ROB. One study [48] had high ROB for selective reporting bias. We were unable to determine the selective reporting bias for twelve studies [15, 26, 28, 31–33, 38, 40, 41, 44, 46, 54] due to insufficient information reported. We detected no other resources of bias for the studies.

### Participant characteristics

The studies involved 7071 patients, with a median of 146 patients per study (range 32–766). Most studies recruited participants with any cancers (n = 21) [15, 23, 25, 26, 29–33, 35, 37–39, 41–45, 47, 50, 52]. Eight studies focused on specific cancers, including lung cancer (n = 3) [27, 28, 34], breast cancer (n = 3) [24, 46, 48], multiple myeloma (n = 1) [36], and leukemia or lymphoma (n = 1) [40]. All studies recruited adult participants, except one study [47] was a pediatric study. Most studies (n = 19) had no limitation to treatments participants received. A few studies focused on participants receiving a particular treatment including chemotherapy (n = 5) [23, 25, 29, 42, 48], surgery (n = 2) [26, 27], immunotherapy (n = 2) [43, 50], and palliative care (n = 1) [32].

### PROM feedback intervention characteristics

Intervention designs varied across the included studies at many aspects, including PROM use, administration approach, audience, content, and feedback message format. We provided summary of intervention characteristics in Table 2. In summary, all researchers developed their interventions to elicit PROM information from patients using validated PROMs. The majority of the interventions collected information about patient symptoms (n = 19) [15, 24, 27, 28, 30–32, 34, 36, 38, 40–42, 44, 46–48, 52] and HRQL (n = 10) [15, 29, 31, 33, 35, 37, 39, 46–48]. Other information included AEs (n = 4) [25, 26, 43, 50] and care needs (n = 4) [31, 33, 35, 48]. Most interventions collected patient information in a non-clinical environment with varying frequencies, including once per visit (n = 9) [15, 34–36, 39–41, 44, 48], once per week (n = 9) [23, 28, 32, 33, 40, 42, 43, 47, 50], twice per week (n = 2) [24, 27],every three months (n = 2) [31, 46], and every two to four weeks (n = 1) [30]. Three interventions [26, 38, 52] were designed to support inpatient care and collect patient information on a daily basis. Three studies [25, 37, 45] reported no or unclear intervention frequency. The majority of the included studies utilized self-administration via web, mobile, and computer applications. Two studies [31, 33] obtained participants’ responses via weekly phone calls by trained monitors.

**Table 2:** Intervention characteristics of the included studies

Study	PROM used	RROM collection frequency	Administration format	Feedback Audience	Feedback content design		
					Report	Treatment advice	Alert
Absolom [23] UK	Author-developed questionnaire collecting symptom presence and severity	Once per week	Self-administrated online questionnaire using PCs or mobile phones	Clinicians and patients	Competed symptom reports were displayed in EHR in real time	Yes	Emails were sent to clinicians for severe symptoms
Anderson [24] USA	Assessments of pain and related symptoms, severity, and barriers	Twice per week	Self-administrated via IVR	Clinicians	Not specified	Not specified	Emails were sent to clinicians when pain level > = 5
Basch [25] USA	12 common symptoms experienced during chemotherapy from the CTCAE	Not specified	Self-administrated via PCs or mobile phones	Clinicians	Patient's symptom burden profiles were provided to clinicians	Not specified	Emails were sent to nurses when a severe or worsening symptom reported
Bryant [26] USA	Adverse events using PRO-CTCAE	Daily	Self-administrated via tablets at post-transplantation	Clinicians	Completed patient reports were immediately sent to nurses	Not specified	Not specified
Cleeland [27] USA	Symptoms using MDASI	Twice per week	Self-administrated via IVR	Clinicians	Not specified	Not specified	Email alerts were sent to clinicians when one or more symptoms met or exceeded a severity threshold
Denis [28] France	Author-developed tool assessing severity of symptoms including appetite loss, fatigue, pain, cough, depression, and breathlessness	Once per week	Self-administrated via a web application	Clinicians	Item scores in a graphical format were sent to clinicians immediately after completion	Not specified	Emails were sent to nurses when criteria were fulfilled based on a dynamic weekly analysis
Detmar [29] The Netherlands	HRQL using QLQ-C30 and SF-36	Once per visit	Self-administrated via a desktop computer in the waiting room	Clinicians and Patients	A paper-based graphical summary profile of patient’s HRQL was provided	Not specified	No alert was provided
Fann [30] USA	ESRA-C questionnaires	Once per 2 to 4 weeks	Self-administrated via a web application	Clinicians and patients	Two-page, color-keyed patient report summary was provided to clinicians before visits	Yes	Verbal notification from research staff was given to clinicians at the time of the visits
Girgis [31] Australia	Anxiety and depression using HADS, HRQL using EORTC version 3, and perceived needs using Supportive Needs Survey – Short Form	Every 3 months	Interviewer-administrated via telephone	Clinicians	A summary page with highlighted concerns and detailed patient scores alongside management strategies via email (txt 1) or mail (txt 2)	Yes	Not specified
Hoekstra [32] The Netherlands	Author-developed questionnaire for prevalence and severity assessment of 10 physical symptoms	Once per week	Self-administrated using a systematic symptom monitoring instrument	Clinicians and patients	Completed questionnaires were provided without a summary	Not specified	Not specified
Kornblith [33] USA	HADS, EORTC QLQ-C30, MOS Social Support Survey	Once per week	Interviewer-administrated via phone call by trained monitors	Clinicians	Not specified	Not specified	Phone calls to oncology nurses when patients scored above cutoff levels within 24 h
Kuo [34] Canada	eLCSS-QL monitoring patient-reported symptoms related to lung cancer disease and treatment	Once per visit	Self-administrated via PDAs	Clinicians	Graphical summaries of eLCSS-QL reports with current scores and changes over time were provided	Not specified	Not specified
Lugtenberg [48] The Netherlands	Standard questionnaire assessing QoL (EORTC QLQ-BR23 & CNB), distress (the NCCN DT), and care needs (open question)	Once per visit	Self-administrated via a web portal or paper-based questionnaire	Clinicians and patients	A graphical summary of patient reports was provided	Not specified	Not specified
McLachlan [35] Australia	CNQ-short form for perceived care needs, EORTC QLQ-C30 for quality of life, and BDI short form for depression measurement	Once per visit	Self-administrated via touch-screen computers	Clinicians	A computer-generated one-page summary of patient reports was provided	Yes	Not specified
Moore [36] Australia	Myeloma Patient Outcome Scale	Once per visit	Not specified	Clinicians	A summary of patient reports was provided	Not specified	Not specified
Nimako [37] UK	EORTC QLQ-C30, EORTC QLQ-LC13	Not specified	Self-administrated paper-based questionnaire in the waiting room	Clinicians	A completed questionnaire was provided without a summary were provided	No	No alert provided
Nipp [38] USA	ESAS-r, PHQ-4	Daily	Self-administrated using tablet PCs	Clinicians	A daily summary and graphical summary of score changes over time were provided	No	An alert was provided whenever a symptom worsened by two or more points or reached an absolute threshold of 4
Nipp [52] USA	ESAS, PHQ-4	Daily	Self-administrated using tablet PCs	Clinicians	A daily summary and graphical summary of score changes over time were provided	No	An alert was provided whenever a symptom worsened by two or more points or reached an absolute threshold of 4
Rosenbloom [39] USA	FACT-G	Once per visit	Txt1: Self-administrated paper-based questionnaire Txt 2: interviewer-administrated paper-based questionnaire	Clinicians	Raw data without summary	Txt 1: No recommendation was provided Txt 2: Items rated as server impairment or worse than expected were highlighted in the reports	Not specified
Ruland [40] Norway	Author-developed assessment tool for cancer-specific symptoms	Once per visit or once per week	Self-administrated via tablet PCs	Clinicians and patients	A printed summary of the assessment was provided	Not specified	Not specified
Ruland [41] USA	Author-developed assessment tool for cancer-specific symptoms	Once per visit	Self-administrated via tablet PCs	Clinicians	A printed summary of the assessment was provided	Not specified	Not specified
Strasser [42] Switzerland	ESAS	Once per week	Self-administrated using handheld PCs	Clinicians	Printed, colored longitudinal monitoring sheets were provided	No	Not specified
Tolstrup [43] Denmark	PRO-CTCAE	Once per week	Self-administrated via tablet PCs	Clinicians and patients	Longitudinal, graphical results were provided	Not specified	Professional healthcare options were provided when patients reported mild or higher adverse events
Trowbridge [44] USA	Author-developed tool assessing pain level, patient satisfaction with regimens, and degrees of pain relief	At baseline visit and four weeks after	Self-administrated via a paper-based questionnaire	Clinicians	A summary sheet of completed patient reports was provided	Not specified	Not specified
van der Hout [45] The Netherlands	Author-developed tool assessing symptom management and HRQL	Not specified	Self-administrated via PCs and mobile phones	Patients	Immediate evidence-based feedback with tailored self-care advice was provided	Yes	Self-help interventions or professional healthcare options were provided when patient scores elevated
Velikova [15] UK	EORTC QLQ-C30 and HADS	Once per visit	Self-administrated via tablet PCs	Clinicians	Longitudinal, graphical summaries of patient reports were provided	No	No
Wheelock [46] USA	SF-36, PHQ-8, and symptom questions modified from the Memorial Symptom Assessment Scale	Every 3 months	Self-administrated via PCs	Clinicians	Completed patient results without longitudinal or graphical summary were immediately sent to clinicians	Not specified	No
Wolfe [47] USA	PQ-MSAS, PEDsQL4.0, and overall sickness question	Once per week or per month	Self-administrated via tablet PCs	Clinicians	Graphical summary profiles of patient reports were provided immediately after completion	Yes	Email alerts were sent to clinicians if patient scores reached predefined thresholds
Zhang [50] China	Author-developed questionnaire of common symptoms based on CTCAE version 5.0	Once per week	Self-administrated via smartphones	Both	Not specified	Yes	Alerts were provided via email, app, and text when a grade 3 or 4 irAE was reported

*BDI – SF*, Beck depression inventory – short form; *CNB*, the care notebook; *CNQ*, care needs questionnaire; *HER*, electronic health record; *eLCSS-QL*, the electronic lung cancer symptom scale; *EORTC QLQ-BR23*, the European Organization for Research and Treatment of Cancer quality of life questionnaire C30; *EORTC QLQ-C30*, the European Organization for Research and Treatment of Cancer quality of life questionnaire – breast cancer 23; *EORTC QLQ-LC13*, the European Organization for Research and Treatment of Cancer quality of life questionnaire – lung cancer 13; *ESAS*, Edmonton symptom assessment system; *ESRA-C*, electronic self-report assessment for cancer; *FACT-G*, the functional assessment of cancer therapy – general; *HADS*, hospital anxiety and depression scale; *HRQL,* health-related quality of life; *IVR*, interactive voice response; *MDASI*, MD Anderson Symptom Inventory; *MOS*, medical outcomes study; *NCCN DT*, national comprehensive cancer network distress thermometer; *PC*, personal computer; *PDA*, Personal digital assistant; *PEDsQL4.0*, the pediatric quality of life inventory 4.0 generic core scales; *PHQ*, patient health questionnaire; *PQ-MSAS*, the PediQUEST memorial symptom assessment scale; *PRO-CTCAE*, patient-reported outcome version of the common terminology criteria for adverse events; *SF-36*: 36-item short-form health survey; *Txt*, treatment group

Main receivers of the PROM feedback were healthcare providers (n = 18) [15, 24–28, 31–38, 40, 44, 46, 52] or both patients and healthcare providers (n = 10) [23, 29, 30, 39, 41–43, 47, 48, 50]. Patients were the only PROM feedback receiver in one study [45]. Eighteen studies utilized graphical summaries to provide patients’ information [15, 23, 25, 28–31, 34–36, 38, 40, 42–45, 48, 52], while five studies [26, 32, 37, 41, 46] presented raw data without any modification. The information feedback formats were unclear for six studies [24, 27, 33, 39, 47, 50]. In addition to patient information, 13 studies [23–25, 27, 28, 30, 33, 38, 39, 43, 45, 50, 52] provided alerts when patient responses reached pre-specified thresholds, and seven studies [23, 30, 31, 35, 45, 50, 52] offered individualized treatment recommendations according to patient responses.

### Effectiveness of PROM feedback interventions by outcomes

#### Health-related quality of life

**Fig. 3:**
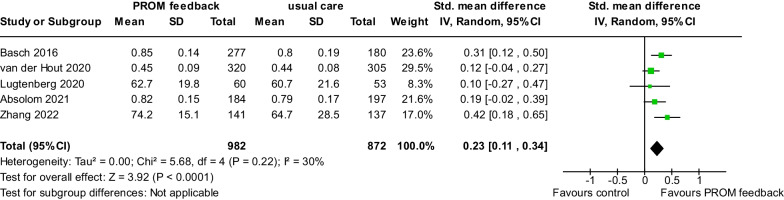
Pooled effects of the patient-reported outcome measure feedback interventions on health-related quality of life improvement

Five studies [23, 25, 45, 48, 50] with 1854 patients evaluated HRQL. Our analysis showed that patients receiving the intervention had a significant improvement in HRQL (Cohen’s d = 0.23, 95% CI 0.11–0.34, *P* < 0.001) compared to those receiving usual care (Fig. 3). Heterogeneity among studies was not substantial (I^2^ = 30%, *P* = 0.22).

It was not possible to include six studies also examining the effect of the intervention on HRQL due to the variations in statistical approaches used and reporting. Of the studies, three studies [15, 42, 47], including 644 participants, found evidence supporting the use of the intervention for HRQL improvement. In contrast, the results of the other three studies [34, 35, 37], involving 672 participants, found that the intervention resulted in no greater improvement in HRQL.

#### Physical, mental, and social functioning

**Fig. 4:**
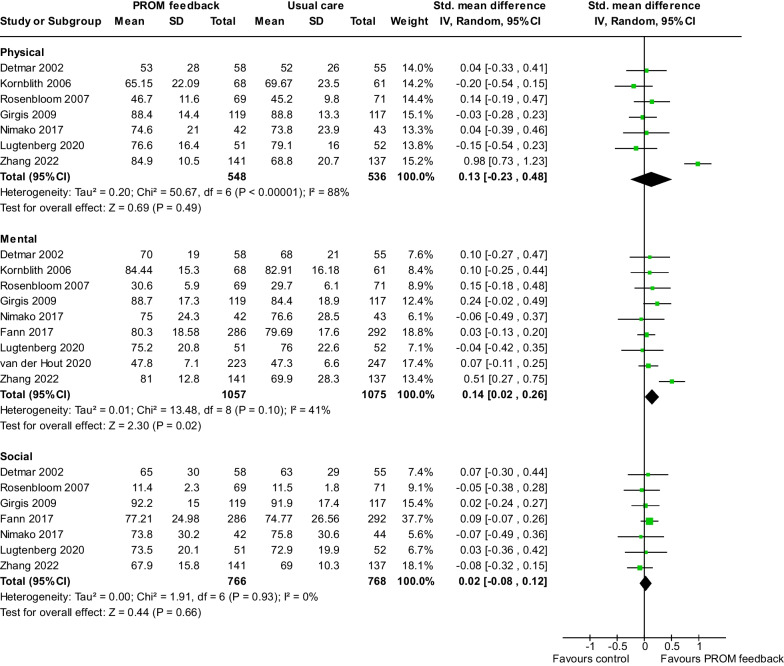
Pooled effects of the patient-reported outcome measure feedback interventions on physical, mental, and social functioning improvement

We identified seven [29, 31, 33, 37, 39, 48, 50], nine [29–31, 33, 37, 39, 45, 48, 50], and seven [29, 31, 33, 37, 39, 48, 50] randomized controlled trials (RCTs) examining physical, mental, and social functioning, respectively. Our meta-analysis revealed that participants had a greater improvement in mental functioning (Cohen’s d = 0.14, 95% CI 0.02–0.26, *P* = 0.02) but not in physical (Cohen’s d = 0.13, 95% CI − 0.23–0.48, *P* = 0.49) and social functioning (Cohen’s d = 0.02, 95% CI − 0.08–0.12, *P* = 0.66) (Fig. 4). We detect a substantial heterogeneity among studies for physical functioning (I^2^ = 88%, *P* < 0.001).

Four studies [23, 35, 42, 52] for functioning were unable to be synthesized due to the lack of mandatory statistics for a meta-analysis. All the studies found that participants in the intervention group experienced no greater improvement in physical functioning. Further, McLachlan et al. [35] showed that the PROM feedback intervention had no improvement in mental and social well-being.

#### Symptom management

For symptom management, we found studies for pain (n = 4) [29, 32, 37, 48], fatigue (n = 3) [32, 37, 48], dyspnea (n = 3) [32, 37, 48], and depression (n = 3) [30, 37, 48] with 400, 284, 285, 806 participants, respectively. Our meta-analyses indicated no improvement in any symptoms for participants receiving the intervention (Pain: Cohen’s d = − 0.01, 95% CI − 0.20–0.19,* P* = 0.96; Fatigue: Cohen’s d = − 0.10, 95% CI − 0.38–0.17, *P* = 0.45; Dyspnea: Cohen’s d = 0.02, 95% CI − 0.21–0.26, *P* = 0.84; and Depression: Cohen’s d = − 0.11, 95% CI − 0.32–0.10, *P* = 0.30) (Additional file 1: Figs. S1–S4). Heterogeneities among the studies for these outcomes were not significant (Pain: I^2^ = 0%, *P* = 0.89; Fatigue: I^2^ = 26%, *P* = 0.26; Dyspnea: I^2^ = 0%, *P* = 0.96; and Depression: I^2^ = 39%, *P* = 0.19).

We also found studies evaluating the effects of the intervention on other symptoms, including nausea (n = 2) [32, 39], anxiety (n = 2) [33, 48], insomnia (n = 2) [32, 48], anorexia (n = 2) [32, 48], constipation (n = 2) [32, 48], diarrhea (n = 2) [32, 48], and cough (n = 1) [32]. However, the pooled effect size estimates for these symptoms may be not reliable due to limited studies available. Overall, participants receiving the intervention showed no greater improvement in any of the individual symptoms in the studies (Additional file 1: Fig. S5).

We were unable to include nine studies [24, 27, 35, 38, 40, 42, 44, 47, 52] which also assessed a variety of symptoms due to missing information. Of them, four studies [38, 40, 42, 47] examined multiple symptoms and consistently reported that the intervention generated a greater reduction in distress. Other symptoms where the intervention sporadically showed effective in the four studies included shortness of breath [38], pain [24, 40], sleep [40], memory [40], worries [40], infection [40] and problems in eating/drinking [40], bowel/bladder [40], and sexuality [40].

Two of the nine studies examined pain severity and reported contradictory results. One study [24] suggested the use of the intervention in pain management, but another earlier study [44] found no greater improvement in pain severity for the intervention group. Two of the nine studies evaluating depression found no benefit of the intervention for the symptom [35, 52]. One study [52] investigated anxiety and detected no greater improvement for the intervention group. Lastly, one study investigated whether the use of the intervention reduced symptom numbers and indicated that the intervention group had 12% fewer symptoms [27]

#### Care process outcomes

**Fig. 5:**
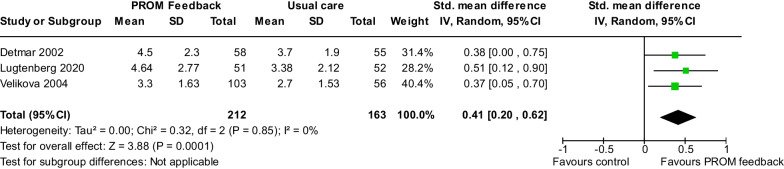
Pooled effects of the patient-reported outcome measure feedback interventions on improving the communication between patients and healthcare providers

*Patient-healthcare provider communication* We identified three studies [15, 29, 48] evaluating self-reported communication between patients and healthcare providers. Our analysis included 375 participants and indicated a moderated improvement in patient-healthcare provider communication (Cohen’s d = 0.41, 95% CI 0.20–0.62, *P* < 0.001) (Fig. 5). Heterogeneity was not significant (I^2^ = 0%, *P* = 0.85).

*Healthcare use* We conducted meta-analyses to examine the intervention effects on the numbers of visits and unplanned hospitalizations. Our meta-analysis for the number of visits involved five studies [23, 25, 28, 43, 50] with 1510 participants and revealed no difference in visit numbers between groups (OR = 1.07, 95% CI 0.76–1.53, *P* = 0.69) (Additional file 1: Fig. S6) and a substantial heterogeneity among studies (I^2^ = 84%, *P* < 0.001). For unplanned hospitalization, our analysis based on three studies with 1286 participants showed no support for the intervention on reducing unplanned hospitalizations (OR = 0.92, 95% CI 0.80–1.06, *P* = 0.27), with no substantial heterogeneity detected (I^2^ = 0%, *P* = 0.50) (Additional file 1: Fig. S6). We could not include Wheelock et al. [46] in the meta-analysis because of missing information. The study reported no difference in the number of visits between groups [46]

*Adverse events* We found three studies [26, 43, 50] evaluating AEs but were unable to conduct a meta-analysis due to missing information. Bryant et al. [26] reported that the intervention group experienced a lower peak of symptom burden (10.4 vs. 14.5, N = 76, *P* = 0.03) within two weeks after hematopoietic stem cell transplantation. Zhang et al. [50] revealed that the intervention did not reduce the occurrence of any immunotherapy-related AEs (irAEs) (Hazard ratio (HR) = 0.63, 95% CI 0.34–1.18, *P* = 0.16) but severe irAEs (HR = 0.51, 95% CI 0.30–0.88, *P* = 0.01). In contrast, Tolstrup and Colleagues [43] found that the intervention did not reduce the number of AEs for individuals receiving immunotherapy (202 vs. 202, N = 146, *P* = 0.56).

**Fig. 6:**
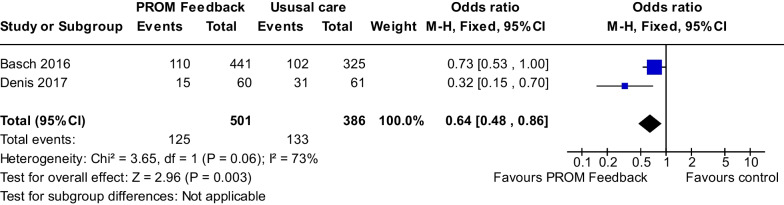
Pooled effects of the patient-reported outcome measure feedback interventions on overall survival improvement

*Overall survival* There were three studies [25, 28, 50] examining OS. Our meta-analysis of two studies involved 887 patients [25, 28] and revealed that the intervention improved patient survival at 1-year (OR = 0.64, 95% CI 0.48–0.86, *P* = 0.003) with substantial heterogeneity among studies presenting (I^2^ = 73%, *P* = 0.06) (Fig. 6). We were unable to include Zhang et al. [49] in the meta-analysis because the outcome of the study was 6-month OS. The study found no survival difference between groups (Hazard ratio = 0.38, 95% CI 0.07–1.99, *P* = 0.28) [50].

## Discussion

We reviewed and quantitatively synthesized results from 29 randomized controlled trials evaluating the effects of PROM feedback on a variety of patient outcomes and care processes. Most interventions were designed to monitor PROMs using self-administered standard instruments via electronic devices and fed patient responses back to healthcare providers to support clinical practices. Our findings suggest that the intervention improved HRQL, mental functioning, patient-healthcare provider communication, and 1-year survival. In contrast, we found unclear evidence on treatment-related AEs and no evidence on outcomes, including physical and social functioning, all symptoms, and numbers of visits and hospitalizations. Our findings are generally consistent with previous reviews reporting inconclusive evidence to support the intervention use in clinical practice for many outcomes [2, 3, 5].

Research suggests that regular collection and monitoring PROMs enable patient-centered care, facilitate better patient-healthcare provider communication, allow identification of unrecognized care needs, and enhance patient symptom management, self-efficacy, and engagement [3, 12, 55]. We found that the intervention had a moderate effect on improving the communication between patients and healthcare providers, consistent with previous studies focusing on similar and other populations [2, 10, 16, 55, 56]. Further, similar to previous reviews [2, 12, 55], we found that providing healthcare providers with PROM feedback slightly improved oncology patients’ HRQL and mental functioning. However, we found unclear evidence supporting the use of the intervention to manage common symptoms for oncology patients. The incongruence may result from the differences in outcome definitions used between the previous and the current studies. Previous studies examined intervention effects on symptoms in general [3, 12], while we advanced the evidence with a greater granularity via examining intervention effects on each symptom. Nevertheless, our approach substantially reduced the number of participants for several symptoms (i.e., cough, nausea, anxiety, etc.) and resulted in findings which, though novel and important, may change as a greater number of studies exploring specific conditions are published. More trials uncovering the effects of the intervention on these outcomes are needed to enable robust evidence synthesis and reliable intervention effect estimates [5].

Survival and treatment-related AEs are critical indicators of life and care quality for oncology patients [3, 12, 25, 28]. We identified limited studies examining these outcomes and considerable ROB among the studies, posing challenges in conducting evidence synthesis. Although we found some studies supporting the use of PROM feedback interventions to reduce AE occurrence and improve 1-year OS, we are unable to recommend the use of the intervention in practices at the current stage based on the narrative synthesis with a limited number of studies. More studies are required to enable additional data on the intervention effects on oncology patient survival and AE management for a more solid evidence evaluation. Moreover, the pathway showing the mechanism of how the intervention leads to improved OS and AE management remains unclear and requires further exploration.

Concerning intervention design, despite some degree of agreement on intervention design (i.e., use of standard instruments and electronic devices for instrument deployment), we found variability in the design of other intervention components among the studies, such as monitoring timing, message receiver, and feedback information content and format. This finding results in an unclear optimal intervention design. Previous studies have demonstrated the importance of identifying effective intervention components to eliminate ineffective intervention components for amplifying intervention adherence, fidelity, and effects [57, 58]. Future studies should explore the relationships between intervention effects and various intervention component designs to enable a guideline supporting future intervention development [2].

Most included studies recruited patients with general cancers and treatments, posing a challenge for a deeper analysis revealing the effectiveness of PROM feedback interventions on specific conditions. The mixed samples may also contribute to the small or non-existence effects of the interventions for many outcomes. Current synthesized evidence, including the present study, suffered from limited numbers of trials targeting one single cancer condition or treatment for conducting subgroup analyses, and thus provided inconclusive information informing implementation of the intervention for care of patients with specific cancer or treatment [2–4]. More research on specific circumstances is needed to enable clinically actionable messages, i.e., the interventions improve irAE management for lung cancer patients receiving immunotherapy. Further, although we did not place language restrictions when searching relevant studies, studies identified and included were all written in English and predominately conducted in English-speaking countries. This may indicate the existence of bias in language and raise concerns about the generalizability of our findings to other countries with diverse language populations worldwide.

In line with previous studies [2, 5, 59], we found ROB in the included studies that future studies can avoid generating unbiased data for robust intervention effect estimates. It is reasonable to use unblinding design given the nature of the interventions. However, we found that most included studies failed to report sufficient information for a determination of bias level in other domains, such as selection, attrition, and reporting bias, as well as intervention contamination. Therefore, we suggest authors of future studies should use standard reporting guidelines (i.e., consolidated standards of reporting trials) to improve trial quality and reporting.

## Conclusions

Our quantitative synthesis of 29 RCTs suggests that the PROM feedback intervention had moderate effects on patient-healthcare provider communication and small effects on HRQL, mental functioning, and 1-year OS improvements. The effects of the intervention on other outcomes are equivocal, and more research is required to enable a more solid evidence evaluation. The ROB among the studies was considerable and obfuscated the real effects of the intervention. Therefore, we concluded that use of the intervention may be effective in improving oncology care but cannot be recommended for clinical practices given the current stage of evidence. Future studies should examine intervention effects by intervention component to reveal optimal intervention design, focus on specific patient conditions to enable granular information, and emphasize thorough reporting to ensure result reproducibility and reliability.

## Supplementary Information

**Additional file 1.** eMethods for database search and Figs S1–5 for pooled effects of patient-reported outcome measure feedback interventions on various symptom reduction.

## Data Availability

All data generated or analyzed during this study are included in this published article and its Additional files.

## Abbreviations

AEs: Adverse events
HRQL: Health-related quality of life
irAEs: Immunotherapy-related adverse events
OS: Overall survival
PROMs: Patient-reported outcome measures
RCTs: Randomized controlled trials
ROB: Risk of bias
RR: Risk ratio
