# Supplementary material for: Effectiveness of routine provision of feedback from patient‐reported outcome measurements for cancer care improvement: a systematic review and meta-analysis
Source: J Patient Rep Outcomes. 2023 Jun 5;7:54. doi: 10.1186/s41687-023-00578-8 (PMC10241766; doi:10.1186/s41687-023-00578-8)
Supplement: Supplementary file 1 — Additional file 1. eMethods for database search and Figs S1–5 for pooled effects of patient-reported outcome measure feedback interventions on various symptom reduction. [file 41687_2023_578_MOESM1_ESM.docx]

**Supplementary Online Content**

**Effectiveness of routine provision of feedback from patient‐reported outcome measurements for cancer care improvement: A systematic review and meta-analysis**

1. eMethods
   1. Search strategies and results for databases
2. eFigures
   1. eFigure 1. Pooled effects of the patient-reported outcome measure feedback interventions on pain reduction.
   2. eFigure 2. Pooled effects of the patient-reported outcome measure feedback interventions on fatigue reduction.
   3. eFigure 3. Pooled effects of the patient-reported outcome measure feedback interventions on dyspnea reduction.
   4. eFigure 4. Pooled effects of the patient-reported outcome measure feedback interventions on depression reduction.
   5. eFigure 5. Pooled effects of the patient-reported outcome measure feedback interventions on the reduction of other symptoms.
   6. eFigure 6. Pooled effects of the patient-reported outcome measure feedback interventions on healthcare use reduction.

**eMethods**

**Search strategies and results for databases**

1. Cochran library

| Date Run: | 2/5/2022 17:37 |  |
| --- | --- | --- |
| ID | Search | Hits |
| #1 | [mh "patient reported outcome measures"] | 972 |
| #2 | (("quality of life" or wellbeing or well-being or QoL or HRQoL or HRQL) near/5 (tool* or questionnaire* or scale* or instrument* or index or indices or measure* or profile* or assess*)):ti,ab | 51187 |
| #3 | self next administ*:ti,ab | 6897 |
| #4 | ((patient* or self) near/2 (report* or apprais* or rate* or rating* or response* or evaluat*)):ti,ab | 113746 |
| #5 | ((patient* or adult*) near/5 complet*):ti,ab | 53605 |
| #6 | self next assess*:ti,ab | 3957 |
| #7 | patient next questionnaire*:ti,ab | 879 |
| #8 | ((function* or health) near/2 status near/2 report*):ti,ab | 439 |
| #9 | (screen* near/2 (tool* or questionnaire* or instrument*)):ti,ab | 3372 |
| #10 | {OR #1-#9} | 206227 |
| #11 | ((physician* or doctor* or nurse* or dentist* or practitioner* or clinician* or team* or anesthetist* or cardiologist* or dentist* or dermatologist* or gastroenterologist* or gp* or geriatrician* or gerontologist* or gynaecologist* or gynecologist* or hematologist* or haematologist* or intensivist* or neurologist* or obstetrician* or oncologist* or paediatrician* or pediatrician* or psychiatrist* or radiologist* or rheumatologist* or surgeon* or urologist*) near/5 (notif* or inform* or disclos* or report* or provid* or result* or recei* or summar* or availab*)):ti,ab | 30634 |
| #12 | (feedback or feed back or "fed back"):ti,ab,kw | 20013 |
| #13 | {or #11-#12} | 48710 |
| #14 | ((routine* or regular*) near/2 ("quality of life" or wellbeing or well-being or QoL or HRQoL or HRQL)):ti,ab | 123 |
| #15 | (#10 and #13) or #14 | 12240 |
| #16 | (tumor* or cancer* or oncolog* or Neoplasm* or toumour*):ti,ab,kw | 227131 |
| #17 | (#15 and #16) with Cochrane Library publication date Between Oct 2021 and Apr 2022 | 146 |

1. Ovid EMBASE

| Date Run: | 2/5/2022 |  |
| --- | --- | --- |
| ID | Search | Hits |
| #1 | Patient Reported Outcome Measures/ | 40675 |
| #2 | ((quality of life or wellbeing or well-being or QoL or HRQoL or HRQL) adj5 (tool? or questionnaire? or scale? or instrument? or index or indices or measure? or profile? or assess*)).ti,ab. | 170291 |
| #3 | self administ*.ti,ab. | 68452 |
| #4 | ((patient? or self) adj2 (report* or apprais* or rate* or rating* or response* or evaluat*)).ti,ab. | 963924 |
| #5 | ((patient? or adult?) adj5 complet*).ti,ab. | 345583 |
| #6 | self-assess*.ti,ab. | 26451 |
| #7 | patient questionnaire?.ti,ab. | 4370 |
| #8 | ((function* or health) adj2 status adj2 report*).ti,ab. | 4167 |
| #9 | (screen* adj2 (tool? or questionnaire? or instrument?)).ti,ab. | 64025 |
| #10 | or/1-9 | 1508443 |
| #11 | ((physician? or doctor? or nurse? or dentist? or practitioner? or clinician? or team? or anesthetist? or cardiologist? or dentist? or dermatologist? or gastroenterologist? or gp? or geriatrician? or gerontologist? or gynaecologist? or gynecologist? or hematologist? or haematologist? or intensivist? or neurologist? or obstetrician? or oncologist? or paediatrician? or pediatrician? or psychiatrist? or radiologist? or rheumatologist? or surgeon? or urologist?) adj5 (notif* or inform* or disclos* or report* or provid* or result* or recei* or summar* or availab*)).ti,ab. | 415269 |
| #12 | feedback/ | 74084 |
| #13 | (feedback or feed back or fed back).ti,ab. | 219204 |
| #14 | or/11-13 | 645460 |
| #15 | ((routine* or regular*) adj2 (quality of life or wellbeing or well-being or QoL or HRQoL or HRQL)).ti,ab. | 629 |
| #16 | (14 and 10) or 15 | 78050 |
| #17 | exp randomized controlled trial/ | 731840 |
| #18 | controlled clinical trial.pt. | 0 |
| #19 | randomi#ed.ti,ab. | 1067928 |
| #20 | placebo.ab. | 342187 |
| #21 | randomly.ti,ab. | 521403 |
| #22 | Clinical Trials as topic.sh. | 2 |
| #23 | trial.ti. | 377795 |
| #24 | exp animals/ not humans/ | 12274965 |
| #25 | or/17-23 | 1814023 |
| #26 | 25 not 24 | 1181088 |
| #27 | 16 and 26 | 7190 |
| #28 | limit 27 to yr="2021 - 2022" | 1360 |
| #29 | cancer.mp. or Neoplasms/ | 4341719 |
| #30 | oncology.mp. or Medical Oncology/ or Radiation Oncology/ or Integrative Oncology/ or Psycho-Oncology/ or Oncology Nursing/ or Surgical Oncology/ | 349859 |
| #31 | (tumor* or cancer* or oncolog* or Neoplasm* or toumour*).ti,ab. | 4488788 |
| #32 | 29 or 30 or 31 | 5358055 |
| #33 | 28 and 32 | 314 |
| #34 | limit 33 to dc=20211005-20220430 | 113 |

1. Ovid MEDLINE

| Date Run: | 2/5/2022 |  |
| --- | --- | --- |
| ID | Search | Hits |
| #1 | Patient Reported Outcome Measures/ | 10307 |
| #2 | ((quality of life or wellbeing or well-being or QoL or HRQoL or HRQL) adj5 (tool? or questionnaire? or scale? or instrument? or index or indices or measure? or profile? or assess*)).ti,ab. | 44572 |
| #3 | self administ*.ti,ab. | 17720 |
| #4 | ((patient? or self) adj2 (report* or apprais* or rate* or rating* or response* or evaluat*)).ti,ab. | 237881 |
| #5 | ((patient? or adult?) adj5 complet*).ti,ab. | 63499 |
| #6 | self-assess*.ti,ab. | 6968 |
| #7 | patient questionnaire?.ti,ab. | 712 |
| #8 | ((function* or health) adj2 status adj2 report*).ti,ab. | 958 |
| #9 | (screen* adj2 (tool? or questionnaire? or instrument?)).ti,ab. | 18033 |
| #10 | or/1-9 | 358893 |
| #11 | ((physician? or doctor? or nurse? or dentist? or practitioner? or clinician? or team? or anesthetist? or cardiologist? or dentist? or dermatologist? or gastroenterologist? or gp? or geriatrician? or gerontologist? or gynaecologist? or gynecologist? or hematologist? or haematologist? or intensivist? or neurologist? or obstetrician? or oncologist? or paediatrician? or pediatrician? or psychiatrist? or radiologist? or rheumatologist? or surgeon? or urologist?) adj5 (notif* or inform* or disclos* or report* or provid* or result* or recei* or summar* or availab*)).ti,ab. | 101608 |
| #12 | feedback/ | 4216 |
| #13 | (feedback or feed back or fed back).ti,ab. | 64956 |
| #14 | or/11-13 | 163806 |
| #15 | ((routine* or regular*) adj2 (quality of life or wellbeing or well-being or QoL or HRQoL or HRQL)).ti,ab. | 204 |
| #16 | (14 and 10) or 15 | 18956 |
| #17 | exp randomized controlled trial/ | 112404 |
| #18 | controlled clinical trial.pt. | 2472 |
| #19 | randomi#ed.ti,ab. | 275000 |
| #20 | placebo.ab. | 57740 |
| #21 | randomly.ti,ab. | 138095 |
| #22 | Clinical Trials as topic.sh. | 15600 |
| #23 | trial.ti. | 108506 |
| #24 | exp animals/ not humans/ | 562923 |
| #25 | or/17-23 | 435263 |
| #26 | 25 not 24 | 409656 |
| #27 | 16 and 26 | 2618 |
| #28 | limit 27 to yr="2021 - 2022" | 996 |
| #29 | cancer.mp. or Neoplasms/ | 779424 |
| #30 | oncology.mp. or Medical Oncology/ or Radiation Oncology/ or Integrative Oncology/ or Psycho-Oncology/ or Oncology Nursing/ or Surgical Oncology/ | 62765 |
| #31 | (tumor* or cancer* or oncolog* or Neoplasm* or toumour*).ti,ab. | 1029618 |
| #32 | 29 or 30 or 31 | 1058612 |
| #33 | 28 and 32 | 180 |
| #34 | limit 33 to dt=20211005-20220430 | 50 |

1. PsycINFO

| Date Run: | 2/5/2022 |  |
| --- | --- | --- |
| ID | Search | Hits |
| #1 | ((quality of life or wellbeing or well-being or QoL or HRQoL or HRQL) adj5 (tool? or questionnaire? or scale? or instrument? or index or indices or measure? or profile? or assess*)).ti,ab. | 35710 |
| #2 | self administ*.ti,ab. | 19923 |
| #3 | ((patient? or self) adj2 (report* or apprais* or rate* or rating* or response* or evaluat*)).ti,ab. | 212316 |
| #4 | ((patient? or adult?) adj5 complet*).ti,ab. | 26277 |
| #5 | self-assess*.ti,ab. | 9333 |
| #6 | patient questionnaire?.ti,ab. | 273 |
| #7 | ((function* or health) adj2 status adj2 report*).ti,ab. | 1101 |
| #8 | (screen* adj2 (tool? or questionnaire? or instrument?)).ti,ab. | 14333 |
| #9 | or/1-8 | 297218 |
| #10 | ((physician? or doctor? or nurse? or dentist? or practitioner? or clinician? or team? or anesthetist? or cardiologist? or dentist? or dermatologist? or gastroenterologist? or gp? or geriatrician? or gerontologist? or gynaecologist? or gynecologist? or hematologist? or haematologist? or intensivist? or neurologist? or obstetrician? or oncologist? or paediatrician? or pediatrician? or psychiatrist? or radiologist? or rheumatologist? or surgeon? or urologist?) adj5 (notif* or inform* or disclos* or report* or provid* or result* or recei* or summar* or availab*)).ti,ab. | 75326 |
| #11 | (feedback or feed back or fed back).ti,ab. | 73651 |
| #12 | feedback/ or "knowledge of results"/ | 21138 |
| #13 | or/10-12 | 148908 |
| #14 | ((routine* or regular*) adj2 (quality of life or wellbeing or well-being or QoL or HRQoL or HRQL)).ti,ab. | 120 |
| #15 | (13 and 9) or 14 | 15788 |
| #16 | exp clinical trial/ | 13322 |
| #17 | random*.ti,ab. | 224956 |
| #18 | ((clinical or control*) adj3 trial*).ti,ab. | 87890 |
| #19 | ((singl* or doubl* or trebl* or tripl*) adj5 (blind* or mask*)).ti,ab. | 28446 |
| #20 | (volunteer* or control group or controls).ti,ab. | 264941 |
| #21 | placebo/ or placebo*.ti,ab. | 42639 |
| #22 | or/16-21 | 500368 |
| #23 | 15 and 22 | 3034 |
| #24 | cancer.mp. or Neoplasms/ | 74526 |
| #25 | oncology.mp. or Medical Oncology/ or Radiation Oncology/ or Integrative Oncology/ or Psycho-Oncology/ or Oncology Nursing/ or Surgical Oncology/ | 12124 |
| #26 | (tumor* or cancer* or oncolog* or Neoplasm* or toumour*).ti,ab. | 82694 |
| #27 | 24 or 25 or 26 | 87378 |
| #28 | 23 and 27 | 265 |
| #29 | limit 28 to up=20211005-20220430 | 4 |

1. CINAHL

| Date Run: | 2/5/2022 |  |
| --- | --- | --- |
| ID | Search | Hits |
| #1 | (quality of life or wellbeing or well-being or QoL or HRQoL or HRQL) N5 (tool? or questionnaire? or scale? or instrument? or index or indices or measure? or profile? or assess*) | 198,412 |
| #2 | self administ* | 21,614 |
| #3 | (patient? or self) N2 (report* or apprais* or rate* or rating* or response* or evaluat*) | 1,473,968 |
| #4 | (patient? or adult?) N5 complet*) | 273,396 |
| #5 | self-assess* | 16,970 |
| #6 | patient questionnaire? | 1,044 |
| #7 | (function* or health) N2 status N2 report*) | 2,214 |
| #8 | (screen* N2 (tool? or questionnaire? or instrument?) | 19,006 |
| #9 | S1 OR S2 OR S3 OR S4 OR S5 OR S6 OR S7 OR S8 | 1,681,187 |
| #10 | (physician? or doctor? or nurse? or dentist? or practitioner? or clinician? or team? or anesthetist? or cardiologist? or dentist? or dermatologist? or gastroenterologist? or gp? or geriatrician? or gerontologist? or gynaecologist? or gynecologist? or hematologist? or haematologist? or intensivist? or neurologist? or obstetrician? or oncologist? or paediatrician? or pediatrician? or psychiatrist? or radiologist? or rheumatologist? or surgeon? or urologist?) N5 (notif* or inform* or disclos* or report* or provid* or result* or recei* or summar* or availab*) | 643,522 |
| #11 | MH "Feedback" | 17,959 |
| #12 | (feedback or feed back or fed back) | 50,135 |
| #13 | S10 OR S11 OR S12 | 679,640 |
| #14 | (routine* or regular*) N2 (quality of life or wellbeing or well-being or QoL or HRQoL or HRQL) | 357 |
| #15 | (MH "clinical trials+") | 343,662 |
| #16 | pt clinical trial | 113,564 |
| #17 | (clin* n25 trial*) | 320,951 |
| #18 | (singl* n25 blind*) or (doubl* n25 blind*) or (trebl* n25 blind*) or (tripl* n25 blind*) | 87,139 |
| #19 | (singl* n25 mask*) or (doubl* n25 mask*) or (trebl* n25 mask*) or (tripl* n25 mask*) | 1,578 |
| #20 | random* or placebo* | 512,037 |
| #21 | (MH "random assignment") | 75,624 |
| #22 | (MH "placebos") | 13,803 |
| #23 | (MH "quantitative studies") | 32,934 |
| #24 | control* or prospective* or volunteer* | 1,935,269 |
| #25 | S15 OR S16 OR S17 OR S18 OR S19 OR S20 OR S21 OR S22 OR S23 OR S24 | 2,211,187 |
| #26 | (MH "Patient-Reported Outcomes") | 4,873 |
| #27 | S9 OR S26 | 1,681,187 |
| #28 | S13 AND S27 | 308,611 |
| #29 | S28 or S14 | 308,866 |
| #30 | S25 AND S29 | 130,538 |
| #31 | tumor* or cancer* or oncolog* or Neoplasm* or toumour* | 801,596 |
| #32 | (MH "Oncology") | 10,226 |
| #33 | S31 OR S32 | 801,596 |
| #34 | S30 AND S33 | 18,835 |
| #35 | ZD (202110* OR 202111* OR 202112* OR 202201* OR 202202* OR 202203* OR 202204* OR "in process") | 962,503 |
| #36 | S34 AND S35 | 2,339 |
| #38 | S36 Limiters - Published Date: 20210101-20221231; Peer Reviewed; Research Article; Randomized Controlled Trials | 98 |

**eFigures**


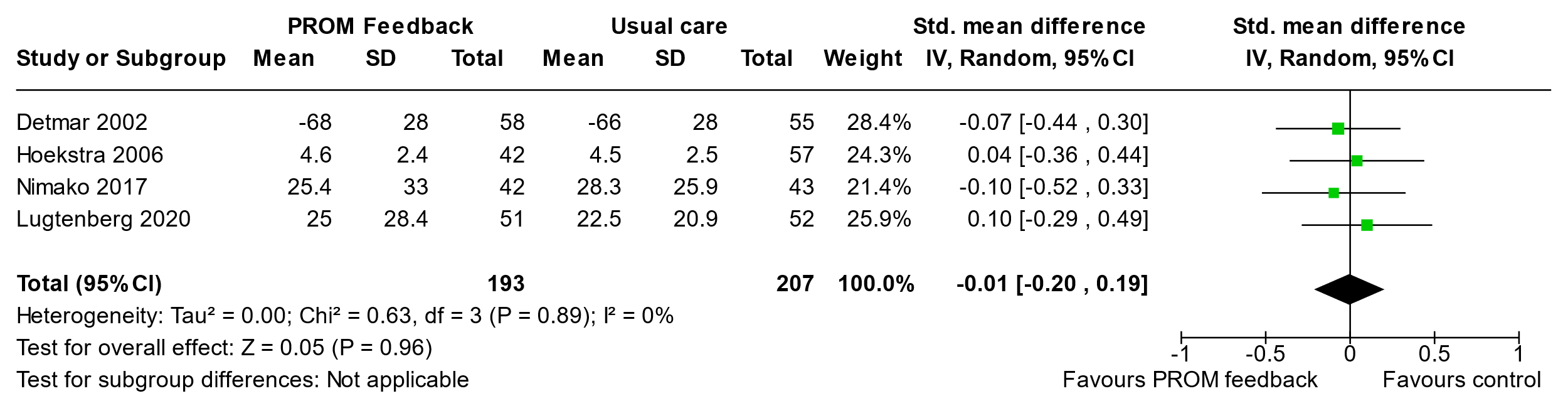


**eFigure 1. Pooled effects of the patient-reported outcome measure feedback interventions on pain reduction.**


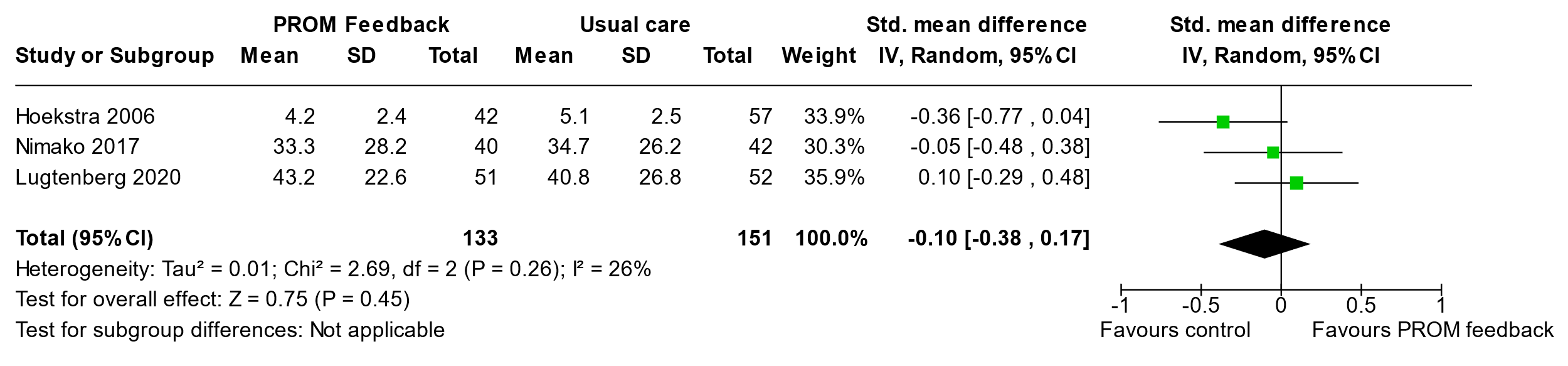


**eFigure 2. Pooled effects of the patient-reported outcome measure feedback interventions on fatigue reduction.**


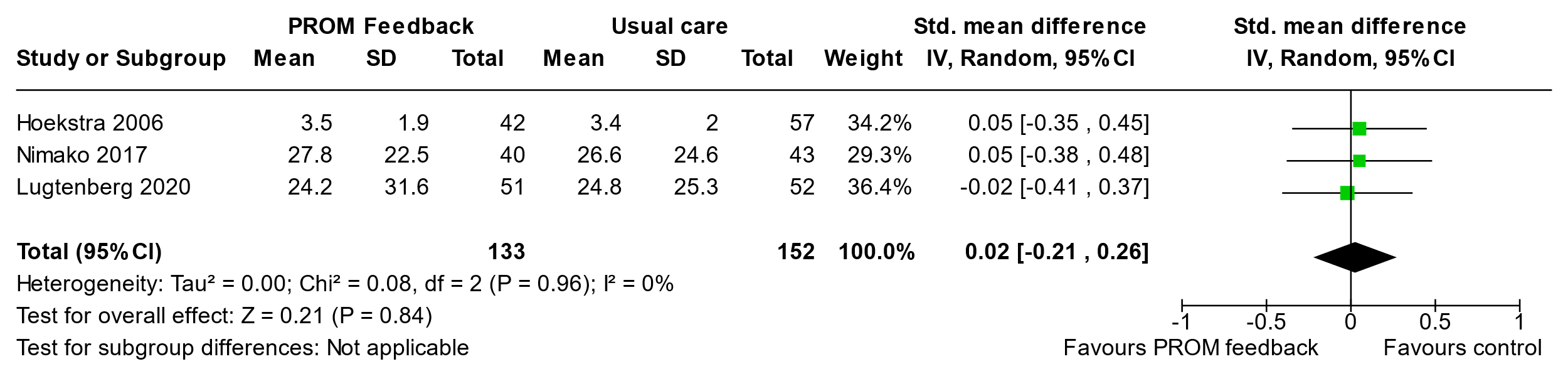


**eFigure 3. Pooled effects of the patient-reported outcome measure feedback interventions on dyspnea reduction.**


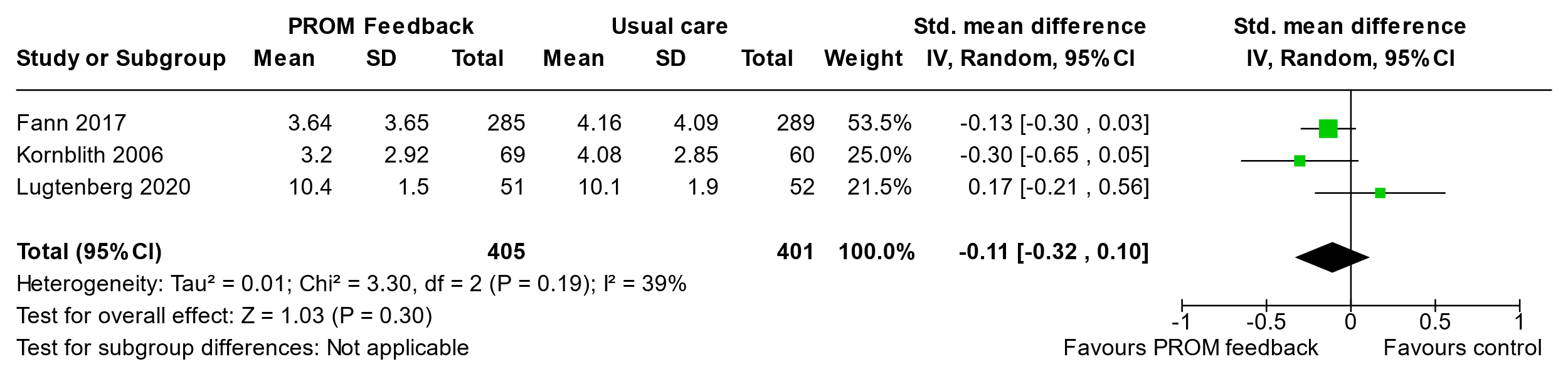


**eFigure 4. Pooled effects of the patient-reported outcome measure feedback interventions on depression reduction.**


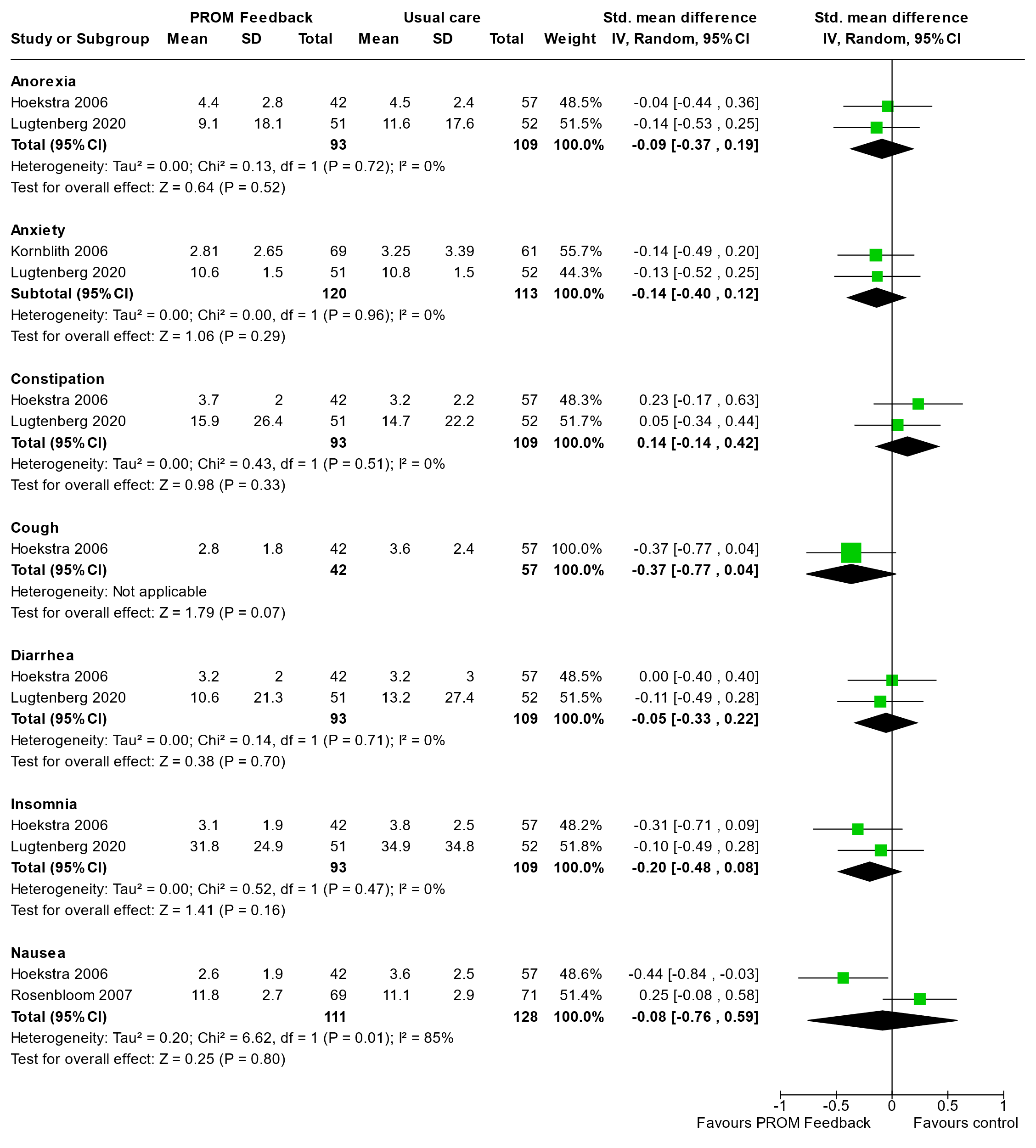


**eFigure 5. Pooled effects of the patient-reported outcome measure feedback interventions on the reduction of other symptoms.**


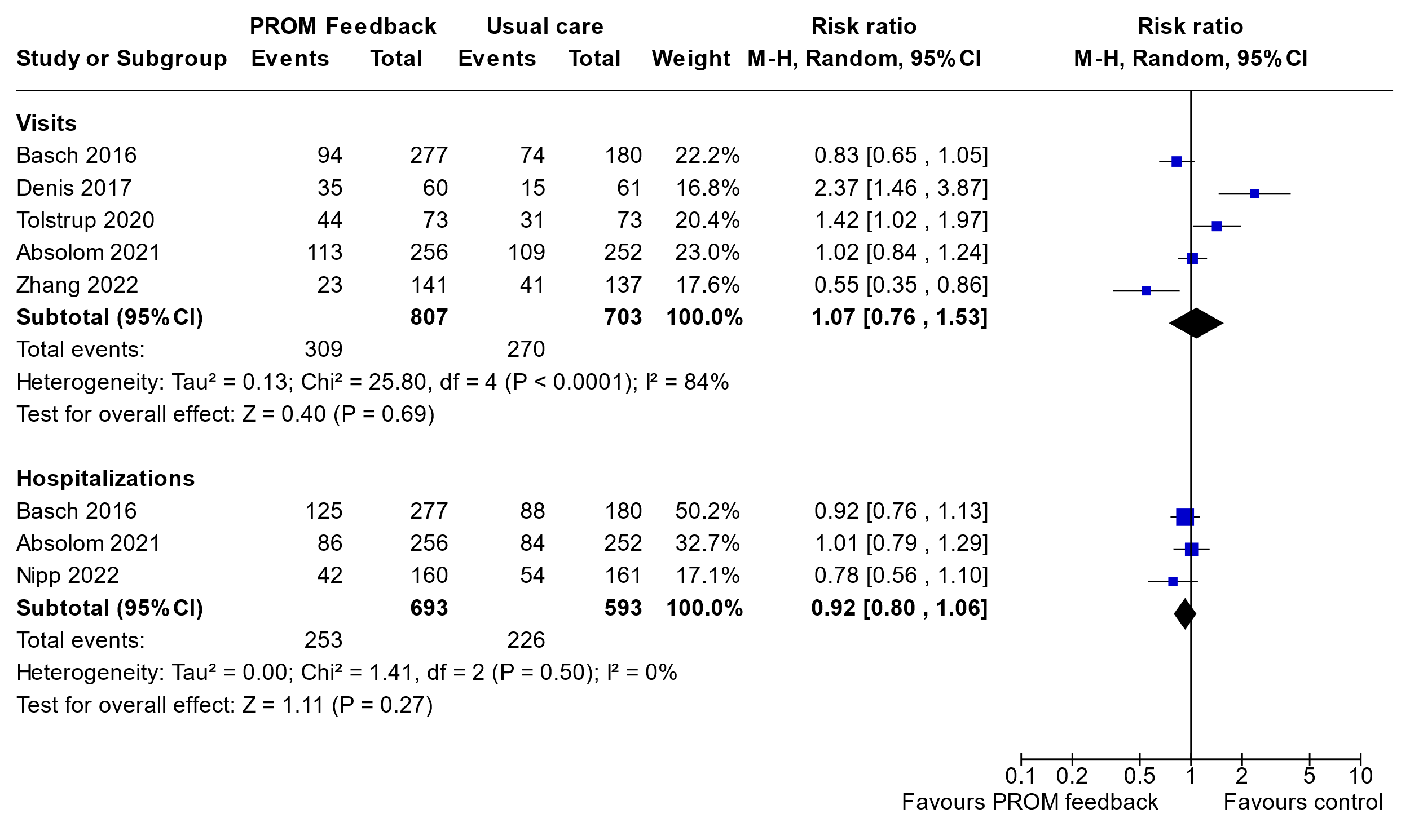


**eFigure 6. Pooled effects of the patient-reported outcome measure feedback interventions on healthcare use reduction.**
